# Editorial Notice

**Published:** 1881-09

**Authors:** 


*Editorial Notice.*—It has been charged by some who are ambitious of making a new departure in journalism that the dental journals are subsidized by manufacturers of dental goods and appliances. If the skirts of all are not clean in this respect, it is a comfort to look over the pages of some of the leading medical journals and see what is going on there. We count in a leading monthly (medical) two long, and ten short editorial "puffs" of drugs and instruments. We suppose it is "business."

